# Supplementary material for: Phenotypic outcomes in Mouse and Human Foxc1 dependent Dandy-Walker cerebellar malformation suggest shared mechanisms
Source: eLife. 2017 Jan 16;6:e20898. doi: 10.7554/eLife.20898 (PMC5271606; doi:10.7554/eLife.20898)
Supplement: Figure 3—source data 1. — DOI: http://dx.doi.org/10.7554/eLife.20898.007 [file elife-20898-fig3-data1.doc]

**Figure 3 – source data 1 – List of control and Dandy-Walker malformation cases listed in the study.**

| Case number | Age | Deletion/Size | Comments |
| --- | --- | --- | --- |
| DW1 | 21 gw | del/dup 17/2.7 Mb | Derivative chr6 from paternal reciprocal translocation t(6;8) |
| DW2 | 19 gw | del/11.5 Mb | *De novo* terminal deletion |
| DW3 | 18 gw | del/dup 6.6/14 Mb | Derivative chr6 from maternal reciprocal translocation t(6;18) |
| DW4 (Fig1C) | 1yr (pn) | del/ >5Mb* | *De novo* |
| DW5 (Fig1E) | 4yr (pn) | del/3.94 Mb | Inheritance unknown |
| Ctrl1 | 23 gw | - | - |
| Ctrl2 | 19 gw | - | - |
| Ctrl3 | 18 gw | - | - |
| Ctrl4 (Fig1A) | 3yr,4mon (pn) | - | - |

*Large visible deletions of chromosome 6p25 by clinical karyotype analysis indicated as >5 Mb, although no molecular studies were performed.

Abbreviations used: DW – Dandy-Walker; gw – gestational weeks; del – deletion; dup – duplication; chr – chromosome; yr – year; mon – months; pn – postnatal.
